# Rifampicin as monotherapy for infections caused by *Staphylococcus aureus*: considerations for not applying ‘breakpoints in brackets’

**DOI:** 10.1093/jac/dkaf392

**Published:** 2025-11-03

**Authors:** Sara E Boyd, Mandy Wootton, Robin A Howe, David M Livermore, Alasdair P Macgowan, Gunnar Kahlmeter

**Affiliations:** David Price Evans Global Health and Infectious Disease Research Group, University of Liverpool, Institute of Systems, Molecular & Integrative Biology, William Henry Duncan Building, Liverpool L7 8TX, UK; National Institute for Health Research Health Protection Research Unit in Healthcare Associated Infections and Antimicrobial Resistance, Imperial College London, Du Cane Road, London W12 0HS, UK; Infection Clinical Academic Group, St. George’s Hospital NHS Foundation Trust, Blackshaw Road, London SW17 0QT, UK; Specialist Antimicrobial Chemotherapy Unit, Public Health Wales, University Hospital of Wales, Cardiff, UK; Specialist Antimicrobial Chemotherapy Unit, Public Health Wales, University Hospital of Wales, Cardiff, UK; Norwich Medical School, University of East Anglia, Floor 2, Bob Champion Research & Educational Building, James Watson Road, Norwich NR4 7UQ, UK; Antimicrobial Reference Laboratory, North Bristol NHS Trust, Westbury-on Trym, Bristol, UK; Clinical Microbiology, Central Hospital, 351 85 Växjö, Sweden

## Abstract

EUCAST introduced ‘breakpoints in brackets’ to warn against the use of specified agents without additional therapeutic measures (EUCAST. Guidance document “EUCAST breakpoints in brackets”. 2021. Available at: www.mic.eucast.org.). Rifampicin is rarely used as monotherapy for *Staphylococcus aureus* infections, yet its breakpoints remain unbracketed. Here, we explore whether this remains appropriate. Although frequent emergent resistance largely precludes rifampicin’s use as monotherapy, this does not meet EUCAST’s core criterion for bracketing—that an agent is inherently ineffective as monotherapy.

In 2021, EUCAST introduced the concept of ‘breakpoints in brackets’ to warn against the use of specific agents ‘without the use of additional therapeutic measures’, particularly where tradition—due to the inherent inadequacy of the agent as monotherapy—is to use it in combination with another active agent, or together with some other intervention.^1^

The breakpoints of rifampicin for *S. aureus* are not bracketed, yet anecdotal evidence suggests that rifampicin is rarely used as monotherapy for staphylococcal infections. In late 2023, we set out to review the extent of data published on this issue and to understand whether a ‘breakpoint in brackets’ should be proposed to EUCAST by the BSAC Standing Committee on Antimicrobial Susceptibility Testing.

A PubMed literature search using both the terms ‘rifampicin’ and ‘*Staphylococcus aureus*’ was conducted on 2 November 2023. Inclusion criteria were *in vitro*, *in vivo* or clinical studies that investigated the use of rifampicin against *S. aureus*. Expert opinion, literature reviews, systematic literature reviews and meta-analyses that appeared relevant were included in case they allowed identification of relevant data or references not captured by the initial search. Exclusion criteria were (i) studies focusing on combination therapy only; (ii) studies investigating novel administration and/or carrier methods, e.g. nanoparticles and microencapsulation; (iii) studies investigating rifampicin in formulations where it is not systemically absorbed or has unpredictable or poorly studied absorption characteristics, e.g. cement spacers; (iv) epidemiological studies; and (v) studies focusing on rifampicin for the treatment of biofilms only. Data were stored and analysed using Microsoft Excel. No ethics approvals were required for this investigation of open-source data.

From the initial search, 545 ‘hits’ were retrieved, and all downloaded to.csv format on the same day. These were then reviewed by one researcher (S.E.B.) and, using the inclusion and exclusion criteria specified above, filtered to 45 abstracts. These abstracts were further reviewed for relevance, and 26 manuscripts were reviewed in full. Of these, two texts were literature reviews based on expert opinion, five were systematic literature reviews (with four including meta-analyses), 17 were experimental studies generating primary data and two were observational studies. These studies were further filtered to 20 full-text manuscripts as some presented no evidence on rifampicin as monotherapy.

The retained papers spanned 45 years and provided diverse findings. Between 1986 and 1987, several studies were published, with various *in vitro* and *in vivo* methodologies, giving promising results for rifampicin monotherapy.^2,3^ An *in vivo* murine thigh model of *S. aureus* infection showed that rifampicin monotherapy produced bacterial kill at 24 h; critically, though, the emergence of resistance was not investigated.^4^ In 1995, Coe *et al.* showed that the combination of ciprofloxacin and rifampicin was at least as efficient as either drug alone for *S. aureus* subcutaneous abscesses in mice, but reported little on the emergence of rifampicin resistance; moreover, (i) only small numbers of mice were investigated,^5^ and (ii) ciprofloxacin monotherapy is not an optimal anti-staphylococcal comparator. In 2016, a neutropenic murine thigh infection model examined the pharmacokinetics/pharmacodynamics (PK/PD) of rifampicin against MSSA, MRSA and VISA.^6^ The authors showed *in vivo* efficacy, best correlated with area under the curve (AUC/MIC) and maximum concentration of drug in serum (*C*_max_/MIC), but acknowledged limitations of their study as its brief (24 h) duration and the lack of investigation of resistance.

However, despite these superficially optimistic data, concerns over the development of resistance with rifampicin monotherapy are evident even from the earliest literature. In 1980, Norden *et al.*^7^ published data from an *in vivo* rabbit osteomyelitis model finding that rifampicin, administered alone for 14 days, was ineffective for sterilizing bones infected with *S. aureus*, possibly owing to the emergence of resistance during treatment, although the authors did not formally assess this. Another study, published in 1981, found that rifampicin monotherapy was rapidly bactericidal for *S. aureus in vitro* and in a murine model of acute and chronic mastitis, but resistance emerged both *in vitro* and *in vivo*.^8^ In 2003, data from time-kill experiments suggested that the rapid emergence of resistant mutants limited the utility of rifampicin as a single agent against *S. aureus*, with regrowth of rifampicin-resistant subpopulations occurring whenever the drug was tested alone.^9^ Unfortunately, rifampicin MICs for the putative mutants at the end of the experiment were not reported.^9^ In time-kill experiments, and in an *in vivo* murine peritonitis model, rifampicin monotherapy resulted in bacterial kill with rapid regrowth over 24 h,^10^ presumably also due to resistance development, but this was not specifically addressed. Rifampicin was observed to inhibit intracellular proliferation of *S. aureus* in a static *in vitro* study,^11^ but rapid acquisition of resistance by alteration of the RNA polymerase encoded by the *rpoB* gene was documented both *in vitro* and *in vivo*.^12^

In short, whilst rifampicin is a demonstrably potent antistaphylococcal antibiotic with good tissue penetration, the swift, high-frequency emergence of resistance limits its utility.^13^ In 2022, a systematic review and meta-analysis identified rifampicin as one of the most effective intracellular agents against *S. aureus*, but observed also that it appears most promising when used in combination to reduce the emergence of resistance.^12^

These data were presented at a recent EUCAST Steering Committee meeting (3–4 September 2024), generating a discussion as to whether ‘breakpoints in brackets’ were merited given that rifampicin is commonly given in combination, due to the risk of resistance development. However, it was decided that brackets were not appropriate as the EUCAST definition for bracketed breakpoints only covers agents that are not efficacious as monotherapy and require the addition of another active agent. Rifampicin may technically be described as efficacious as monotherapy, as per the *in vitro* and *in vivo* studies discussed earlier, but is rarely used in this capacity, and so does not qualify for a bracketed breakpoint.

It is therefore important to be aware, when selecting antimicrobial therapy, that just because breakpoints for an agent are not in brackets, monotherapy may not be suitable. A bracketed breakpoint indicates that the agent is simply not efficacious without the use of additional therapeutic measures, but does not address the risk of rapid drug resistance development. Further research is needed investigating rifampicin (i) *in vivo*, (ii) in dynamic *in vitro* PK/PD models and (iii) in clinical studies, designed specifically to address the question of what combination therapy and dosing thereof best reduces the emergence of drug resistance.

## Transparency declarations

S. E. B.: research support from Roche Pharma, educational support from Advanz Pharma, consultancy and/or speaker fees from Meiji, Sumitovant, Shionogi, the UK Clinical Pharmacy Association and bioMérieux. D. M. L.: advisory boards or *ad hoc* consultancy, AdjuTec, AstraZeneca, Centauri, GenPax, GSK, Lipovation, Meiji, Paion, Shionogi, Sumitovant, Thermo Fisher and Wockhardt; paid lectures, bioMérieux, Pfizer, Shionogi and Zuellig Pharma; and relevant shareholdings or options, GenPax, GSK, Merck & Co., Inc. and Revvity amounting to less than 10% of portfolio value. D. M. L. also has shareholdings in Arecor, Celadon Pharma, Genedrive, Genincode, Probiotix, Rua Life Sciences, SkinBiotherapeutics and VericiDx (all with research/products pertinent to medicines or diagnostics) through Celadon Pharma and Genedrive, but has no authority to trade these holdings directly. A. P. M.: research grants/activities with Merck, Shionogi, InfectoPharm, GSK, Roche, BioVersys, iFAST, Oxford Drug Design, Bicycle Therapeutics, PACE, JPIAMR and NIHR. The other authors have nothing to declare.
